# Efficiency and Quality of Data Collection Among Public Mental Health Surveys Conducted During the COVID-19 Pandemic: Systematic Review

**DOI:** 10.2196/25118

**Published:** 2021-02-10

**Authors:** Yu-Hsuan Lin, Chung-Yen Chen, Shiow-Ing Wu

**Affiliations:** 1 Institute of Population Health Sciences National Health Research Institutes Miaoli County Taiwan; 2 Department of Psychiatry National Taiwan University Hospital Taipei Taiwan; 3 Department of Psychiatry College of Medicine, National Taiwan University Taipei Taiwan; 4 Institute of Health Behaviors and Community Sciences College of Public Health, National Taiwan University Taipei Taiwan; 5 Department of Environmental and Occupational Medicine National Taiwan University Hospital Taipei Taiwan; 6 Institute of Environmental and Occupational Health Sciences College of Public Health, National Taiwan University Taipei Taiwan

**Keywords:** COVID-19, mental health, Newcastle-Ottawa Scale, review, data collection, survey, surveillance, literature, research

## Abstract

**Background:**

The World Health Organization has recognized the importance of assessing population-level mental health during the COVID-19 pandemic. During a global crisis such as the COVID-19 pandemic, a timely surveillance method is urgently needed to track the impact on public mental health.

**Objective:**

This brief systematic review focused on the efficiency and quality of data collection of studies conducted during the COVID-19 pandemic.

**Methods:**

We searched the PubMed database using the following search strings: ((COVID-19) OR (SARS-CoV-2)) AND ((Mental health) OR (psychological) OR (psychiatry)). We screened the titles, abstracts, and texts of the published papers to exclude irrelevant studies. We used the Newcastle-Ottawa Scale to evaluate the quality of each research paper.

**Results:**

Our search yielded 37 relevant mental health surveys of the general public that were conducted during the COVID-19 pandemic, as of July 10, 2020. All these public mental health surveys were cross-sectional in design, and the journals efficiently made these articles available online in an average of 18.7 (range 1-64) days from the date they were received. The average duration of recruitment periods was 9.2 (range 2-35) days, and the average sample size was 5137 (range 100-56,679). However, 73% (27/37) of the selected studies had Newcastle-Ottawa Scale scores of <3 points, which suggests that these studies are of very low quality for inclusion in a meta-analysis.

**Conclusions:**

The studies examined in this systematic review used an efficient data collection method, but there was a high risk of bias, in general, among the existing public mental health surveys. Therefore, following recommendations to avoid selection bias, or employing novel methodologies considering both a longitudinal design and high temporal resolution, would help provide a strong basis for the formation of national mental health policies.

## Introduction

The World Health Organization has recognized the importance of assessing population-level mental health during the COVID-19 pandemic. More than 463 articles on the mental health impact of COVID-19 have been published in 2020, and several more mental health surveys are underway [1]. Most of these studies evaluated the psychiatric symptoms among the general public and health care workers, and some others evaluated patients with COVID-19 or those with psychiatric disorders [2]. In a global crisis such as the COVID-19 pandemic, time-sensitive policy decision-making underscores the importance of fostering an agile empirical approach that can monitor population-level mental health in a timely manner. Any change in the mental health impact of COVID-19 is likely to be dynamic. For example, the 20% decrease in suicide rate in Japan observed during the early stage of the pandemic seemed to have reversed in August 2020, when a 7.7% rise was reported [3]. Therefore, a timely surveillance method is urgently needed to track the impact of COVID-19 on public mental health. This brief systematic review focused on studies on the general public conducted during the COVID-19 pandemic and analyzed the efficiency and quality of data collection in the existing literature.

## Methods

**Figure 1:**
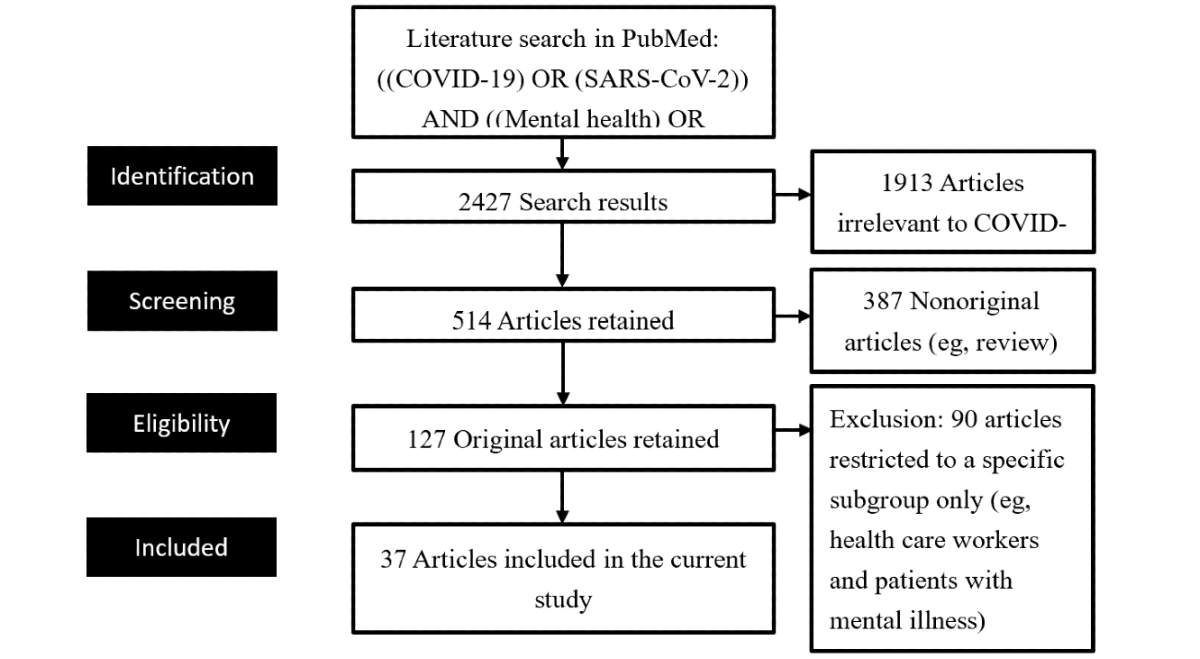
PRISMA (Preferred Reporting Items for Systematic Reviews and Meta-Analyses) flowchart of article selection for the systematic review.

We used the following search strings to select relevant articles in the PubMed database: ((COVID-19) OR (SARS-CoV-2)) AND ((Mental health) OR (psychological) OR (psychiatry)). We screened the titles, abstracts, and texts of the published articles to exclude irrelevant studies. We also excluded articles published on preprint platforms such as bioRxiv, because we were unable to analyze the time between the receipt of the articles and their availability online. A PRISMA (Preferred Reporting Items for Systematic Reviews and Meta-Analyses) flow diagram detailing the study retrieval process is shown in Figure 1.

We used the Newcastle-Ottawa Scale to evaluate the quality of each research article. The Newcastle-Ottawa Scale includes four criteria for selection bias, with scores ranging between 0 and 5; scores <3 points are regarded as high risk for selection bias in a cross-sectional study [1,4]. The four quality assessment criteria were as follows: sample representativeness (1 point), predetermined and satisfactory sample size (1 point), comparability between respondents and nonrespondents (1 point), and ascertainment of the measurement tool (2 points for the validated measurement tool; 1 point for the nonvalidated measurement tool, but the tool is available or described).

## Results

In all, we found 37 relevant mental health surveys of the general public during the COVID-19 pandemic by searching the PubMed database, as of July 10, 2020. All these public mental health survey studies examined had a cross-sectional study design. The journals efficiently made these articles available online in an average of 18.7 (range 1-64) days from the date they were received. The average duration of recruitment period of these studies was 9.2 (range 2-35) days, and the average sample size was 5137 (range 100-56,679). The correlation coefficient (*r*=0.164, *P*=.40) between these durations and the corresponding sample sizes suggested that the samples in the studies selected from the existing literature were collected in an efficient manner; even larger sample sizes did not require proportionately longer to collect the data. Although there was a potential publication bias in the recruitment or data collection period, it is noteworthy that approximately 92% (34/37) of the studies reported the use of a web-based or an app-based survey, thereby promoting the efficiency of large-scale data collection, especially during the COVID-19 pandemic.

Unlike most studies on health care workers, which have a low risk of selection bias [1], our review showed that 73% (27/37) of the selected studies evaluating the mental health impact of COVID-19 on the general public had Newcastle-Ottawa Scale scores of <3 points, which suggests that these studies were of very low quality to be considered for inclusion in a meta-analysis. All of the selected studies met the criterion of validated measurement tools, but only 8 studies met the criterion of sample representativeness, 5 met the criterion of justified sample size, and 1 met the criterion of comparability between respondents and nonrespondents (Table 1).

**Table 1:** Public mental health surveys conducted during the COVID-19 pandemic.

Publication	Sample size (n)	Recruitment period (2020)	Duration of recruitment period (days)	Duration between article submission and online publication (days)	Newcastle-Ottawa Quality Assessment Scale scores					
					Sample representativeness	Sample size	Nonrespondents	Ascertainment of exposure	Total score	
Ahmed et al [5]	1074	—^a^	—	32	0	0	0	2	2	
Bryan et al [6]	10,625	March 18 to April 4	18	—	1	1	0	2	4	
Cao et al [7]	7143	—	—	6	1	0	1	2	4	
Castelli et al [8]	1321	March 19 to April 5	18	—	0	0	0	2	2	
Choi et al [9]	500	April 24 to May 3	10	18	1	1	0	2	4	
Cortes-Alvarez et al [10]	1105	March 30 to April 5	7	—	0	0	0	2	2	
Fitzpatrick et al [11]	10,368	March 23-29	7	—	1	0	0	2	3	
Gao et al [12]	4827	January 31 to February 2	3	43	0	0	0	2	2	
Huang and Zhao [13]	7236	February 3-17	15	23	0	0	0	2	2	
Lei et al [14]	1593	February 4-10	7	25	0	0	0	2	2	
Li et al [15]	4607	February 2-9	8	—	0	0	0	2	2	
Liang et al [16]	584	January 30 to unknown	—	—	0	0	0	2	2	
Liu et al [17]	285	January 30 to February 8	10	1	0	0	0	2	2	
Ma et al [18]	728	April 9-30	22	—	0	0	0	2	2	
Mazza et al [19]	2766	March 18-22	5	19	0	0	0	2	2	
McGinty et al [20]	1468	April 7-13	7	—	1	0	0	2	3	
Moccia et al [21]	500	April 10-13	4	5	1	1	0	2	4	
Moghanibashi-Mansourieh [22]	10,754	March 1-9	9	22	0	0	0	2	2	
Naser et al [23]	4126	March 22-28	7	64	0	1	0	2	3	
Ozdin and Bayrak Özdin [24]	343	April 14-16	3	—	0	1	0	2	3	
Qiu et al [25]	52,730	January 31 to February 10	11	7	0	0	0	2	2	
Rehman et al [26]	403	April 3-6	4	49	0	0	0	2	2	
Roy et al [27]	662	March 22-24	3	7	0	0	0	1	1	
Smith et al [28]	932	March 17 to unknown	—	25	0	0	0	2	2	
Shi et al [29]	56,679	February 28 to March 11	13	—	0	0	0	2	2	
Sonderskov et al [30]	2458	March 31 to April 6	7	13	1	0	0	2	3	
Tan et al [31]	673	February 24-25	2	6	0	0	0	2	2	
Tian et al [32]	1060	January 31 to February 2	3	15	0	0	0	2	2	
Ustun [33]	1115	March 23 to April 3	12	—	0	0	0	2	2	
Verma and Mishra [34]	354	April 4-14	11	—	0	0	0	2	2	
**Wang et al [35]**										
	1210	January 31 to February 2	3	4	0	0	0	2	2	
	861	February 28 to March 1								
Xiao et al [36]	170	—	—	11	0	0	0	2	2	
Yuan et al [37]	939	—	—	22	0	0	0	2	2	
Yuan et al [38]	100	—	—	—	0	0	0	2	2	
Zhang and Ma [39]	263	January 28 to February 5	9	20	0	0	0	2	2	
Zhang et al [40]	369	February 20-21	2	10	1	0	0	2	3	
Zhu et al [41]	2279	February 12 to March 17	35	1	0	0	0	2	2	

^a‑^Data not available.

## Discussion

The general public surveys examined in this review most commonly used the web-based format to promote efficiency of large-scale data collection, especially in the countries with significant COVID-19 outbreaks that had introduced social distancing or lockdown measures. However, the web-based survey approach has an inherent methodological limitation to show the comparability between respondents and nonrespondents. The sample representativeness of the target population is critical for selection bias. Web-based survey platforms using sampling methods could avoid such selection bias. Two published surveys [6,11] used Qualtrics Panels, a web-based survey platform that uses quota sampling methods to identify participants who meet a given study’s eligibility criteria, with a target to recruit a sample that is demographically similar to the 2010 United States census distributions for age, sex, and race or ethnicity (with a ±10% variation). Qualtrics Panels maintains a database of several million US residents who have volunteered to participate in periodic survey-based research. Another study [20] used AmeriSpeak, a probability-based research panel designed to be representative of the adult US population. AmeriSpeak data is sourced from National Opinion Research Center’s area probability sample and a US postal service address–based sample covering approximately 97% of all US households. In this national survey study [20], a higher proportion (13.6%) of the US adult sample reported symptoms of serious psychological distress in 2020 than in 2018 (3.9%).

The limitations of cross-sectional studies should also be noted when interpreting the role of the COVID-19 outbreak on the mental health of the general public. These epidemiological studies identified general risk factors during the COVID-19 outbreak, rather than the specific impacts of COVID-19 on the mental health of the public. For example, a cross-sectional study of 4827 participants [12] showed that lower education levels and higher social media exposure result in a high risk of individuals developing depressive symptoms, but these findings did not suggest that COVID-19 had an impact on the public’s mental health. There was only one longitudinal study [35], which conducted two-wave surveys during the initial COVID-19 outbreak (ie, January 31 to February 2, 2020) and during the peak 4 weeks later (ie, February 28 to March 1). Although this study recruited 1210 and 861 participants within 3 days by using a web-based survey approach, no significant longitudinal changes in stress, anxiety, or depression levels were observed. These findings might result from a lack of baseline data, few follow-ups with the same respondents (333/1210, 27.52%), or insufficient temporal resolution to detect mental health changes. Only one study compared the prevalence of symptoms of serious psychological distress before and during the COVID-19 pandemic by using an identical measure. This survey, which used AmeriSpeak for sample recruitment, showed that the prevalence of serious psychological distress symptoms among US adults was higher during the COVID-19 pandemic in 2020 than that reported in the 2018 National Health Interview Survey [20]. However, this study did not monitor the changes in population-level mental health through the unfolding pandemic. Timely mental health survey studies with a high temporal resolution, such as internet search data (eg, Google Trends) [42] and sentiment analysis on social media (eg, Weibo posts) [43], are warranted in the future to monitor the long-term impacts of the fast-moving COVID-19 outbreak.

In conclusion, this systematic review found that the data collection methods used in the public mental health surveys in the existing literature were efficient but generally had a high risk of bias. Therefore, following recommendations to avoid selection bias, or to apply novel methodologies considering both longitudinal design and high temporal resolution, would help provide a strong basis for the formation of national mental health policies.
